# Spatial Variability in Condition of Southern Rock Lobsters (*Jasus edwardsii*) at the Start of the Tasmanian Fishing Season

**DOI:** 10.1371/journal.pone.0166343

**Published:** 2016-11-15

**Authors:** Tania Mendo, Cedric Simon, Bridget Green, Caleb Gardner

**Affiliations:** Institute for Marine and Antarctic Studies, Fisheries and Aquaculture Centre, University of Tasmania, Private Bag 49, Hobart 7001, Australia; University of Waikato, NEW ZEALAND

## Abstract

The southern rock lobster (*Jasus edwardsii*) industry in Australia favours red lobsters, which are usually caught in shallow waters, over paler (brindle) lobsters. This preference is driven partly by the Chinese market, where red is associated with luck and prosperity, and additionally, by the widely held perception within the industry that brindles have greater mortality rates during out of water transport than reds. Limited scientific evidence supports these industry observations; however, these studies did not evaluate the initial condition of lobsters. This study aimed first, to determine which variables better describe condition in *J*. *edwardsii* and second, to compare condition among lobsters in several sites around Tasmania at the typical time of high transport mortality. Male lobsters were collected from the South West, South East, East and North coast of Tasmania in late November/December 2014, which correspond to the start of the Tasmanian fishing season. A comprehensive condition assessment was applied by measuring tissue proximal composition, Brix index, Total Haemocyte Count, pH, haemocyanin and another 16 haemolymph parameters of interest. A useful framework to compare condition in *J*. *edwardsii* was established by first, using Brix index as a measure of nutritional condition, second, using pH, magnesium, and bicarbonate to evaluate differences in physiological condition and finally, using THC counts as a proxy for lobster health condition. Lobsters from different sites had different nutritional, physiological and health condition, consistent with industry observations, however our results indicate that some red shallow water lobsters exhibited poorer nutritional and health condition, while some deep water brindle lobsters were in good condition. Differences in condition could not be directly associated to catch depth of lobsters and was related to other spatially discrete factors which sometimes vary over distances <3 km.

## Introduction

The annual export of southern rock lobsters (*Jasus edwardsii*) from Tasmania, Victoria and South Australia to markets in Asia is currently 3,500–4,000 tonnes with a gross revenue of approximately AUD200 million [1]. This species is harvested from a range of depths: premium lobsters tend to be caught in shallow-water areas (usually < 30 m depth) and are bright red in colour (‘reds’) while lobsters harvested from deep-water areas are paler (‘brindle’, ‘speckled’ or ‘pales’) [2]. Lobster exporters favour red lobsters and sometimes pay AUD$10/kg or 15% more for red lobsters compared to brindle. This preference is driven in part by the Chinese market, where there is a cultural association of red with luck, happiness, and prosperity [3]. Additionally, there is a widely held perception within the industry that lobsters from deeper waters (brindle) have greater mortality rates during out of water transport. This is reportedly most critical around the time of season openings in November and associated mainly with male lobsters that moult just before this opening.

Scientific evidence supporting these industry observations is limited and contradictory ([1, 4, 5]. In addition, some of these studies did not evaluate initial condition of lobsters, making it difficult to assess which factors were most important in explaining observed differences in survival rates. We suggest that in order to determine which factors are affecting survival of lobster, first we need to establish a useful framework to assess condition. Once this framework is in place, we can evaluate the spatial variability in condition among lobsters to further inform experimental designs attempting to evaluate differences in survival.

Haemolymph protein concentration, and associated techniques to estimate it via refractometry, have been used as the main non-destructive index (e.g., Refractive index, Brix index) for measuring “nutritional condition” in lobsters [6–8]. Processors generally don’t assess nutritional condition but rather rely on observations of vitality (e.g. tail flipping and antenna response) to assess condition prior to live-holding in anticipation for live transport to markets. Vitality is strongly affected by stressors associated with live transport including capture and handling by fishing gear and crew, exposure to varying temperatures, oxygen availability and physical damage [9]. It is therefore biased towards a measure of “physiological condition”, which can be readily reversible, if the physiological disturbance is within the homeostatic capability of the lobsters, or it can be non-reversible ultimately leading to mortality [9]. The study of crustacean haemolymph biochemistry, which includes a large range of organic and inorganic constituents, provides a measure of physiological condition and can provide useful information to the degree of stress in wild population, during live-holding and live transport [6, 10]. Haemocyanin is the oxygen-carrying protein in the haemolymph, and hypoxia or lack of oxygen can result in an increase in haemocyanin in crustaceans [11]. Finally, total haemocyte count (THC) has been used as an indicator of health, with healthy lobsters having higher THC counts than moribund lobsters [12]. THC has been found to change rapidly in response to hypoxia and handling during transport (see review in [13]).

Blood biochemistry panels developed for veterinary use provide the opportunity of analysing a wide range of haemolymph constituents from the same animal [14, 15] and were used in this study to determine which variables better describe nutritional, physiological, and health condition in *J*. *edwardsii* and second, to assess differences in condition among lobsters from several sites around Tasmania at the typical time of high transport mortality. A comprehensive condition assessment was applied by measuring tissue proximal composition, Brix index, THC, pH, haemocyanin and another 16 haemolymph parameters of interest.

## Methods

**Fig 1:**
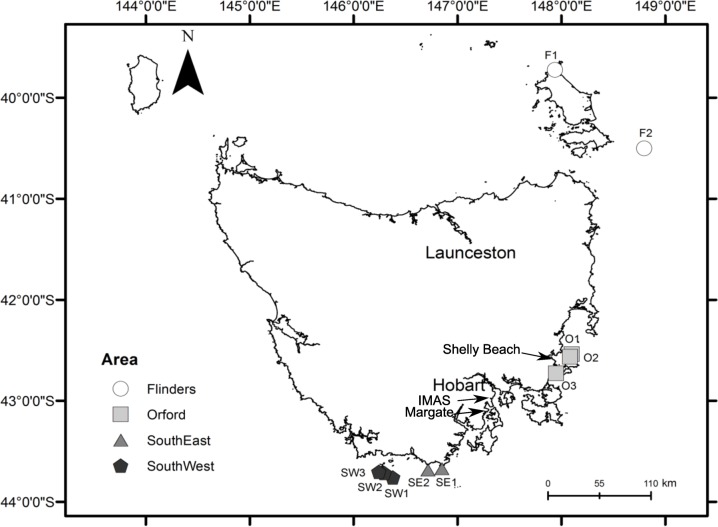
Map of sampling sites at four different sampling areas (Flinders, Orford, South East and South West) and sites (F1, F2, O1, O2, O3, SE1, SE2, SW1, SW2, and SW3) around Tasmania.

Male lobsters were collected from the South West, South East, East and North coasts of Tasmania in late November/December 2014 with two to three sites sampled from each area (Fig 1). The commercial fishery sets gear during both the day and the night but in this case all samples were collected from an overnight fishing shot to reduce handling variation. Each shot involved the setting of 5–30 lobster traps (pots) by a commercial fishing vessel. The study was conducted under the Authority of the Department of Primary Industries, Parks, Water and Environment (DPIPWE) permit No. 14211, 14212 and 14217. After capture, lobsters were held in tanks in each commercial vessel for 1–3 days, and then transported by car in crates covered with wet hessian sacks to the IMAS aquaculture laboratory facilities in Taroona, Tasmania (Table 1). Lobsters were held in crates submerged in a 1000 l tank with free-flowing seawater and sampled within the first 2 hours after arrival to the facilities. Lobsters from the South East and South West were all brindle in colouration and were caught in waters ranging from 84–99 m depth. On the East coast, near Orford, brindle and red lobsters were found at different depths (20–40 m depth) and around Flinders Island, only red lobsters were caught at sites with 25 m depth (Table 1). Colour was assigned following Chandrapavan et al. [2] as either ‘Red’ or ‘Brindle (a combination of brindle and white). Basic data on each lobster (carapace length and weight) was recorded and the distal half of one pleopod was cut and kept to determine the moulting stage, following Musgrove [16]].

**Table 1:** Lobster colour and average depth of sites located in different areas around Tasmania. Landing port locations are shown in Fig 1.

Area	Site	Average time in holding tank (d)	Landing port	Driving distance from port (h)	Average depth (m)	Colour
Flinders	F1	2	Margate	0.5	25	Red
	F2	2	Margate	0.5	25	Red
Orford	O1	0.5	Shelly Beach	1.5	40	Brindle
	O2	0.5	Shelly Beach	1.5	20	Red
	O3	0.5	Shelly Beach	1.5	20	Red
South East	SE1	3	Margate	0.5	89	Brindle
	SE2	3	Margate	0.5	88	Brindle
South West	SW1	1	Margate	0.5	96	Brindle
	SW2	1	Margate	0.5	99	Brindle
	SW3	1	Margate	0.5	84	Brindle

In order to determine if differences in nutritional condition of male lobsters differed amongst areas around Tasmania, lobsters (*n* = 156) from all sites in each area were sampled non-destructively for Brix index. A total of 100 μl of whole haemolymph was added directly to an automatic temperature compensated digital refractometer to measure the Brix index (%) (Hanna HI96801, Hanna Instruments, Australia). The refractometer was zeroed using deionised water. Biochemical composition analyses of tissues were used to calculate various biochemical indices of nutritional condition and to analyse the relationship between these and haemolymph biochemistry parameters as well as the Brix index for this species. A subsample of 54 male lobsters from all areas were destructively sampled immediately after taking the haemolymph sample by bringing them to a chill-coma on ice and were kept frozen until dissection at −40°C. Lobsters were weighed and the hepatopancreas (HP) and abdominal muscle (AM; entire muscle mass of the tail including extension into the cephalothorax) dissected out and weighed (± 0.01 g).

Dry matter content of the AM tissue was analysed by weight change following freeze-drying to a constant weight as follows:

$${AM}_{DM}\;(\%)={AM}_{tissue\;freeze-dried\;weight}\frac{{AM}_{DW}}{{AM}_{tissue\;wet\;weight}}({AM}_{WW})\;x\;100$$

Total lipid (TL) in the HP (HP_TL_) was determined by the method of Bligh and Dyer (1959) using chloroform: methanol: water (1: 1: 0.9) and expressed in mg g^-1^ of hepatopancreas wet tissue (HP_WW_) for 40 lobsters.

### Haemolymph biochemistry

Haemolymph (2.5 ml) was collected from the base of the 5th leg of 78 lobsters collected at sites F2, O1, O2, O3, SE1, SE2, SW2, and SW3 using a 3 ml syringe and 26 G Terumo needle pre-chilled on crushed ice. Whole haemolymph pH was measured using a Testo 205 temperature compensated pH probe. A total of 200 μl of whole haemolymph was extracted from each lobster and added to 200 μl Baker’s Formol Calcium anticoagulant (4% Formaldehyde, 2% NaCl, 1% calcium acetate) to estimate total haemocyte count (THC). Samples were placed on a slide haemocytometer with grids of 0.0625 mm^2^ and three pictures of different grids were recorded using 100x magnification. THC was estimated using the software Image J (v1.48c) and calculated with the following equation:

THC(cellsperml)=ŷxdilutionfactorxvolumefactor

Where ŷ = average count of cells per grid, dilution factor of sample (generally = 2), and the volume factor of a grid was defined by the haemocytometer as 80000.

Two aliquots of 1000 μl were extracted from a subsample of 52 lobsters originating from sites F2, O2, O3, SE2, SW2, and SW3, centrifuged at 3,000 × g for 4 min, the haemocyte-free plasma removed into clean cryovials, and snap-frozen in liquid nitrogen. Samples were then stored at − 80°C. The first aliquot was shipped frozen to Diagnostic Services at the Atlantic Veterinary College, University of Prince Edward Island, Canada, and analysed using a Cobas c501 automated biochemistry analyzer (Roche Diagnostics Corporation, Indianapolis, IN, USA) for a full blood profile consisting of the electrolytes (mmol l^-1^) sodium (Na), chloride (Cl), potassium (K), magnesium (Mg) and bicarbonate (bicarb); minerals (mmol l^-1^) calcium (Ca) and phosphorus (P); metabolites (mmol l^-1^) glucose (Gluc), lactate (Lact), cholesterol (Chol), triglyceride (Trig), total protein in the haemolymph (TP, in g l^-1^), urea, and uric acid (Uric, in μmol l^-1^). The second aliquot was used as a backup and to determine haemocyanin. Twenty μl of plasma were diluted 30 times in deionised water for analysis of oxygenated haemocyanin (OxyHc) at 334 nm [17] in a Synergy HT plate reader. An extinction coefficient of E = 17.26 was used for OxyHc determination [17].

### Statistical analyses

To assess whether Brix index was a good indicator of nutritional condition of *J*. *edwardsii*, linear regressions were fitted to model the relationship between the Brix index and AM_DM_, DG_TL_, and total protein in the haemolymph (TP), respectively. Spatial differences in the Brix index (as an indicator of nutritional condition in lobsters), were assessed using a Nested ANOVA due to the hierarchical nature of the sampling design (sites inside areas) [18]. The main factor was the sampling Area (South West, South East, Flinders, and Orford) and the nested factor was the site inside each area. A nested ANOVA was also used in two sites (South West and South East) where there was enough replication (4–15 lobsters per pot) to determine if there was any variation in lobster Brix index that could be explained at the level of the sampling pot.

Haemolymph biochemistry data were explored using factor analysis, which is a statistical method that groups variables that are highly correlated to each other and is used to simplify the interpretation of trends down to only a few factors (i.e., groups) [19]. Haemolymph biochemistry parameters of lobsters tend to strongly correlate because the concentrations measured are ultimately dependent on the individual lobster haemolymph volume [7]. To assist with interpretation, factors were rotated by an orthogonal transformation (Varimax rotation). Factors with eigenvalues greater than 1 were retained. Variables were considered to contribute to a factor if the factor loading was greater than or equal to 0.5. Variables that did not load on any factor were removed from the analysis [19]. One variable from each factor (except Factor 2, associated with nutritional condition) was chosen to give a complete representation of physiological condition in *J*. *edwardsii*, based on replicability of sampling or existing literature available for discussion. These three variables were: pH, bicarb, and Mg. This step was essential as collinearity between variables is a problem when using MANOVA (see subsequent section) and not including highly correlated (redundant) variables will lessen this negative impact [18].

Differences in physiological condition among sites were assessed using a MANOVA test. A sequential Bonferroni (Holm’s method) was used to adjust the p-values from the pairwise contrasts among the sites [18]. The Shapiro-Wilk Multivariate Normality Test was used to assess multivariate normality and the Box’s M test was used to test homogeneity of covariance matrices using a p value <0.005 to reject the null hypothesis [20]. A canonical discriminant analysis followed the MANOVA to identify the variables that explained the differences in the centroid means for each site. Statistical analysis was conducted using the R software package [21].

THC means were compared using an ANOVA, followed by a Tukey’s post-hoc test to determine if there were any differences amongst sites or lobster colour. Normality of residuals was assessed visually by plotting the residuals. Homogeneity of variances was assessed using the Bartlett’s test [22]. Tukey’s post hoc tests were used to determine which sampling sites differed [23]. If the homogeneity of variance assumption was violated, a generalised least squares (GLS) model was fitted [24], using the varIdent variance structure to allow a different pattern of spread of residuals to vary per site [24].

## Results

Lobsters from Flinders were significantly smaller and weighed less on average than lobsters collected from the other three areas (Table 2). Microscopic examination of pleopods confirmed that all lobsters were in intermoult stage.

**Table 2:** Size, weight and biochemistry profiles of lobsters from different sites around Tasmania. Data are expressed as mean (SE). Note different sample sizes for haemolymph biochemistry profiles.

	F2	O2	O3	SE2	SW2	SW3
Sample size (n)	12	14	9	18	28	47
Carapace length (mm)	85.5 (3.86)	104.36 (1.14)	105.63 (0.82)	99.05 (1.58)	98.79 (1.39)	101.08 (2.48)
Weight (g)	318.47 (46.2)	548.54 (15.49)	604.25 (13.25)	479.4 (25.87)	482.28 (34.79)	507.73 (36.38)
pH	7.63 (0.06)	7.37 (0.14)	7.14 (0.02)	7.68 (0.12)	7.57 (0.03)	7.66 (0.08)
Sample size (n)	7	18	6	4	7	10
Cl (mmol L^-1^)	495.5 (12.03)	524.14 (28.03)	504 (9.53)	538.5 (4.01)	510.6 (10.5)	524.25 (23.8)
Mg (mmol L^-1^)	9.1 (0.28)	10.68 (0.61)	6.58 (0.03)	13.28 (1.54)	11.1 (0.62)	10.49 (0.69)
Bicarb (mmol L^-1^)	10.55 (1.75)	8.11 (1.07)	3.9 (1.97)	8.75 (1.1)	6.5 (1.76)	5.06 (1.93)
P (mmol L^-1^)	0.63 (0.19)	2.36 (0.74)	2.93 (0.16)	1.03 (0.26)	1.48 (0.21)	1.15 (0.22)
Urea (mmol L^-1^)	0.05 (0.02)	0.11 (0.03)	0.15 (0.02)	0.1 (0)	0.08 (0.02)	0.1 (0.02)
Gluc (mmol L^-1^)	0.97 (0.13)	3.06 (1.07)	2.63 (0.14)	1.85 (0.13)	1.12 (0.14)	1.05 (0.09)
Lact (mmol L^-1^)	0.8 (0.4)	11.02 (3.68)	13.39 (0.35)	1.64 (0.66)	2.51 (0.58)	2.06 (0.71)
Uric (mmol L^-1^)	41.17 (13.33)	123.43 (41.74)	60.75 (7.25)	35.5 (2.94)	31.2 (6.65)	47.25 (8.43)
OxyHc (mmol L^-1^)	0.49 (0.02)	0.61 (0.09)	0.74 (0.09)	0.75 (0.02)	0.43 (0.06)	0.68 (0.03)
Ca (mmol L^-1^)	14.1 (0.42)	16.36 (0.86)	15.89 (0.29)	17.4 (0.35)	16.15 (0.3)	16.41 (0.37)
Chol (mmol L^-1^)	0.23 (0.03)	0.53 (0.13)	0.72 (0.06)	0.45 (0.01)	0.44 (0.03)	0.42 (0.04)
Trig (mmol L^-1^)	0.38 (0.16)	0.49 (0.13)	0.92 (0.13)	0.51 (0.02)	0.48 (0.03)	0.58 (0.05)
TP (g L^-1^)	36.33 (5.63)	57 (11.48)	67 (2.91)	72.5 (2.4)	67.2 (3.81)	68.63 (4.75)
Na (mmol L^-1^)	543.83 (44.76)	533.57 (21.56)	510.75 (8.9)	541.5 (2.4)	519 (6.56)	525.75 (18.75)
K (mmol L^-1^)	9.57 (0.54)	8.1 (0.44)	8.1 (0.16)	9 (0.32)	8.46 (0.53)	8.89 (0.73)

### Brix Index as an indicator of nutritional condition in *Jasus edwardsii*

**Fig 2:**
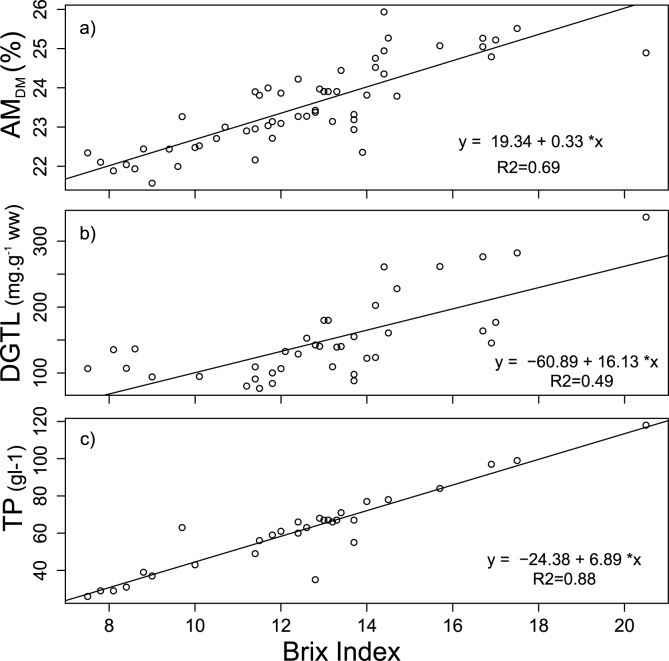
**Relationship between Brix index and a) AM**_**DM**_ **(abdominal muscle dry matter content), b) HP**_**TL**_ **(hepatopancreas total lipid in wet weight), and c) TP (total haemolymph protein).** Regression lines and corresponding equations are shown.

The Brix index was significantly related to HP_TL_ (F = 37.59, df = 1, 37, p<0.001), AM_DM_, (F = 118.22, df = 1,53, p<0.001) and TP (F = 211.51, df = 1, 29, p<0.001) (Fig 2) and was therefore considered a good indicator of nutritional condition in lobsters. The Brix index correlated best with TP (r^2^ = 0.88), followed by abdominal muscle dry matter content (r^2^ = 0.69), and hepatopancreas lipid reserves (r^2^ = 0.49).

### Brix index as a non-invasive spatial indicator of nutritional condition

**Fig 3:**
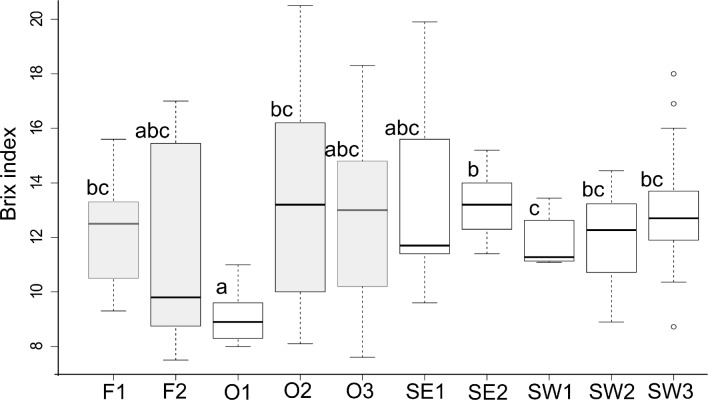
Boxplot of Brix index amongst sites around Tasmania. The boxes enclose data falling between the 1^st^ and 3^rd^ quartile and the lines in bold represent the median in each location. The bars indicate the 95% confidence intervals of the median. Data points falling outside these ranges are plotted individually. Different letters above the bars indicate significant differences between sites. Grey boxes represent sites with shallow water red lobsters (except site O1, with shallow water brindle lobsters), while white boxes represent sites with deep water brindle lobsters.

There was significant variation in the Brix index between the replicate sites within each sampling area but there was no significant difference in the Brix index among areas (Table 3). Significant differences between sites were driven mainly by one site in Orford (O1, north of Maria Island), which had a lower mean Brix index compared to the South West (Fig 3). This site was deeper (40 m) than the other two sites at Orford (20 m) and separated from its nearest site (O2) by 3.2 km (Table 1, Fig 1). Lobsters from this site had a brindle colouration. Also one site in the South East (SE2, 88 m depth) differed in the Brix index compared to one site in the South West (SW1, 96 m depth). At the level of sampling pot, the nutritional condition of lobsters was very similar (F = 2.79, df = 11,55, p = 0.66).

**Table 3:** Variation in Brix index within and between areas (Nested ANOVA).

Source of variation	df	MS	F	p
Area	3	5.944	1.106	0.348
Site (Area)	6	16.06	2.99	0.0081
Residual	143	5.37		

### Physiological condition

**Fig 4:**
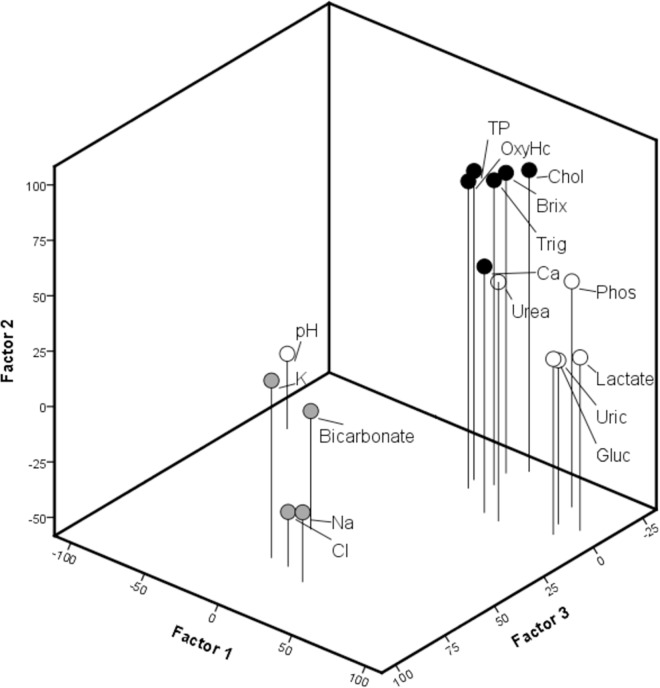
Rotated factor loadings for haemolymph biochemistry data. Factor 1: white circles; factor 2: black circles; factor 3: grey circles.

Factor analysis of the haemolymph biochemistry data resulted in four main factors explaining 75% of the cumulative variation (Fig 4): Factor 1 accounted for 45% of the variation, and included highly correlated plasma parameters such as pH, P, Gluc, Lact, urea and uric acid. Factor 2 accounted for 14% of the variation and included Brix index, OxyHc, Ca, Chol, trig, and TP, and was therefore deemed as a better indicator of nutritional condition. Factor 3 accounted for 10% of the variation and included Na, K and Cl and bicarb; and Factor 4 accounted for 7% of the variation and included Mg. The last aforementioned factor could not be included in Fig 4.

**Fig 5:**
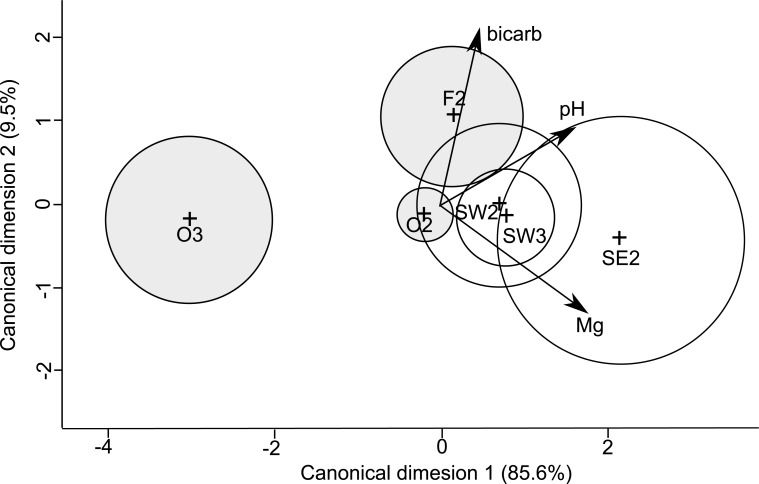
The centroid means for each of the sites plotted in the first two canonical discriminant dimensions. The direction and length of the vectors show the strength and nature of the correlation with each variable and the canonical discriminant axes. The percent values for each axes is the percentage of variability among the centroid means explained by each of the two axes. Grey circles represent sites with red shallow water lobsters, while white circles represent sites with brindle deep water lobsters.

Physiological condition was different among sites (Fig 5; F = 3.01, df = 5,44, p<0.001), which was driven by the levels of pH and Mg in the haemolymph. Lobsters from the South West and the South East were associated with higher concentrations of Mg in the haemolymph, while lobsters from Orford were associated with lower pH. Pairwise contrasts showed significant differences between site SE2 and O2 (F = 8.56, df = 1,20, p = 0.01), SE2 and O3 (F = 23.59, df = 1,8, p = 0.01), SW2 and O3 (F = 14.81, df = 1,10, p = 0.01), SW3 and O3 (F = 13.14, df = 1,14, p = 0.01), O2 and O3 (F = 12.07, df = 1,22, p = 0.006), and, F2 and O3 (F = 11.14, df = 1,11, p = 0.01). Shallow water lobsters were associated with lower pH values while brindle deep water lobsters were associated with greater concentrations of Mg in the haemolymph.

### Health condition

**Fig 6:**
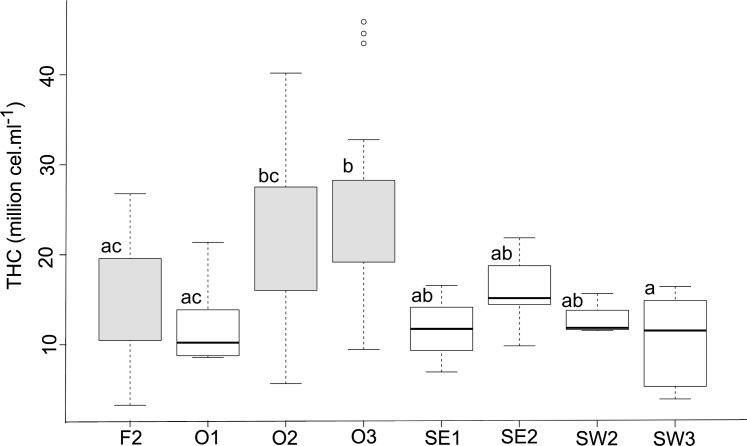
Boxplot of THC counts amongst sites around Tasmania. For explanation on boxplots refer to Fig 3. Different letters above the bars indicate significant differences between sites. Grey boxes represent sites with red shallow water lobsters, while white boxes represent sites with brindle deep water lobsters.

The average THC counts differed among sites (Fig 6; F = 4.16, df = 7,67, p<0.001). Site O3 had a greater THC count than sites F2, O1 and SW3.

## Discussion

By evaluating a wide array of haemolymph constituents from the same animal, we were able to establish a useful framework to compare nutritional, physiological and health condition of the southern rock lobster, *Jasus edwardsii*. This framework can be summarised as a combination of first, Brix Index as a measure of nutritional condition, second, the evaluation of pH, magnesium, and bicarbonate to evaluate differences in physiological condition and finally, as a proxy for lobster health condition, total haemocyte counts (THC). We then used this framework to demonstrate that male lobsters originating from different sites in Tasmania differed in nutritional, physiological and health condition. Nevertheless, these differences were not always evident between lobsters of different catch depths or colouration. For example, brindle lobsters from one site at Orford (O1, 40 m depth) were in poorer nutritional condition than red lobsters from the other two sites at Orford (O2 and O3; ~20 m depth), however, no such differences were detected between these two sites and brindle lobsters from South East and South West Tasmania (SE1, SE2, SW1, SW2, SW3; ~90 m depth). Similarly, while overall differences in THC between shallow water red and deep water brindle lobsters were detected, these differences were not always consistent at the level of site (e.g. differences in health condition were not evident between red lobsters located in F2, O2, and O3, compared to brindle lobsters collected in SE1, SE2, and SW2). Furthermore, THC for both brindle and red lobsters had overall high THC consistent with good health. THC below 4 million cells/ml have previously been reported as an indication of poor condition or health in *P*. *cygnus* lobsters [12]. Only one lobster from SW2, one from SW3 and two from O2 had THC below this level of 4 million cells.ml^-1^. Nevertheless, comparisons between studies need to be made with caution, as THC varies between species and due to a range of environmental factors (emersion, temperature) [13]. There is a lack of specific information on baseline THC for *J*. *edwardsii* which warrants future research. Our observations strongly suggest that site-specific characteristics (i.e. density of conspecifics, predator risk, prey availability), are driving the differences in lobster condition in Tasmania. The presence of sites with lobsters in poor nutritional and health condition (e.g. site O1) is consistent with observations by the industry in Tasmania that lobsters from some areas have greater mortality rates during out of water transport. However our results indicate that the effect of site is complicated, and is not sufficiently explained by site depth alone.

The Brix index correlated strongly to haemolymph total protein as well as to both hepatopancreas and abdominal muscle tissue composition in our study. Previous studies have also demonstrated refractometry (e.g., Brix index, refractive index, density) to be an easy and affordable field method to estimate nutritional condition of crustaceans including lobsters [7, 15, 25]. In our study, the Brix index correlated better to abdominal muscle dry matter content than hepatopancreas lipid reserves suggesting it was a better indicator of the nutritional condition associated with the tail muscle density (i.e., ‘meat content’) than lipid stored in the hepatopancreas for energy. The hepatopancreas of decapod crustaceans is involved in lipid synthesis and storage, and responds rapidly to changes related to dietary stress and moulting [7, 26]. In contrast, percent dry matter of abdominal muscle in *J*. *edwardsii* showed no significant change after 28 days of starvation [26] suggesting that this measurement is useful to understand longer term dietary changes experienced by the animal. Therefore, the Brix Index seems to be a better indicator of long term than short term energy reserves in *J*. *edwardsii*.

Variation in nutritional condition based on the non-invasive Brix index assessment was larger between sites within broad areas of the coast than between broad areas or within pots. For example, lobsters collected from the deeper Orford site (O1), which were brindle-coloured, were in lower nutritional condition as measured by the Brix index than in other shallower sites of Orford and deeper sites in SE and SW. This suggests that if measurements were taken from lobsters to guide harvesting, data would need to be collected across small (fine) spatial scales. The close relationship between the Brix index and nutritional condition, combined with the ease of sampling suggests that further sampling of this index through the course of routine observer catch sampling for stock assessment, which is already conducted at fine spatial scales in Tasmania and samples lobsters caught in about 12000 pots per year (average value during the period 2012–2016), could be worthwhile to guide harvesting patterns. These data could conceivably also help in fisheries assessment, for example to identify years with unusually high or low growth, which would affect the productivity of the stock independently of recruitment. Linking the Brix index to condition of lobsters is complex however as the relationship may vary from year to year depending on changes in factors such as water temperature, productivity and timing of the moult cycle. For example, in our sampling, male lobsters across all areas on the whole were in better nutritional condition (i.e., within intermoult stage) in late November-December 2014 (average Brix index of 12.2) than previously noted from similar areas in November 2007 and 2008 (RI of 1.34–1.346 = Brix index of 6.5–10.0) [6, 27]. Additionally, the Brix index in this study correlated well to tail dry matter content, a likely proxy for meat yield, as the Brix index correlates well to meat yield in American lobster *Homarus americanus* [28]. Measuring the Brix index non-invasively could allow differentiation of product on the basis of meat yield which may be important in some markets. Fishers could possibly estimate meat yield of catch from only a few pots given the low variation in nutritional condition observed at the pot level.

Lobsters collected from deeper areas (South East and South West) were associated with greater values of magnesium, compared to lobsters from shallower areas (Orford or Flinders). Similarly, Chandrapavan et al [6] found greater where levels of magnesium in lobsters from deep areas in southern Tasmania than lobsters collected from a shallow reef at Taroona. Additionally, Chandrapavan et al [6] found that when deep water lobsters with higher magnesium were translocated to shallow reefs, within a year their magnesium levels were reduced to the same levels as resident shallow water lobsters. As levels of activity and the concentration of magnesium in the haemolymph are inversely related in crustaceans [29, 30], these results suggest that lobsters in deeper areas have lower levels of activity than lobsters in shallower areas.

In general, the haemolymph of lobsters originating from Orford had lower pH than in those from other areas. This is more than likely related to the different times exposed to air during transportation (Table 1). Lobsters collected from Orford were exposed to a three-fold difference in emersion time compared with the other areas. A significant physiological issue during emersion is the alteration of the internal acid-base balance as the gills collapse in air so that the surface area for gas exchange is reduced [31, 32]. This reduces the uptake of oxygen and the elimination of carbon dioxide from the haemolymph. Reduction in haemolymph oxygen leads to anaerobic metabolism and accumulation of lactate in the tissues [10]. The relationship between pH and lactate concentration in the haemolymph for *J*. *edwardsii* showed that the blood pH initial drop from 7.8 to 7.3 is associated with limited lactate build up (<5mmol l-1) but decreases in pH < 7.2 are characterised by much higher lactate levels >10 mmol l^-1^ linked to much greater reliance on anaerobic metabolism and are therefore of greater biological significance (See S1 Appendix). Nevertheless, differences in pH observed in this study could not be associated with survival issues after limited road transport, as all lobsters recovered to normal pH levels (7.5–7.8) in the holding tanks including those from Orford which had the lowest pH on arrival. A previous study showed pH to be a poor indicator of survival pre- and post-emersion [5]. Instead the pre-emersion concentration of haemolymph bicarbonate and oxy-haemocyanin were useful predictors of lobster survival and a model was proposed to calculate the risk of dying. THC, bicarbonate and oxy-haemocyanin were compared after 40 hours of emersion and no significant differences were found between pre-emersion and after emersion (Table 3). As this strongly suggests that these variables were not affected by the much shorter emersion time during this study (a maximum of 1.5 hours), we explored this model [5] further to predict the vulnerability to emersion of lobsters from each site using the average values for bicarbonate and oxy-haemocyanin at each site (Table 2). The predicted risk of dying was greater for site SW2, followed by O2, F2, SE2, SW3, and finally O3 (See S2 Appendix). Again, differences in vulnerability to emersion were independent of catch depth or carapace colour and rather depended on site.

## Supporting Information

S1 Appendix Relationship between haemolymph pH and lactate (mmol L^-1^). (DOCX)

S2 Appendix Average risk of dying and associated standard errors per site. (DOCX)
