# Supplementary figures and images for: Spatial Variability in Condition of Southern Rock Lobsters (Jasus edwardsii) at the Start of the Tasmanian Fishing Season
Source: PLoS One. 2016 Nov 15;11(11):e0166343. doi: 10.1371/journal.pone.0166343 (PMC5112911; doi:10.1371/journal.pone.0166343)

Supplementary material 1.


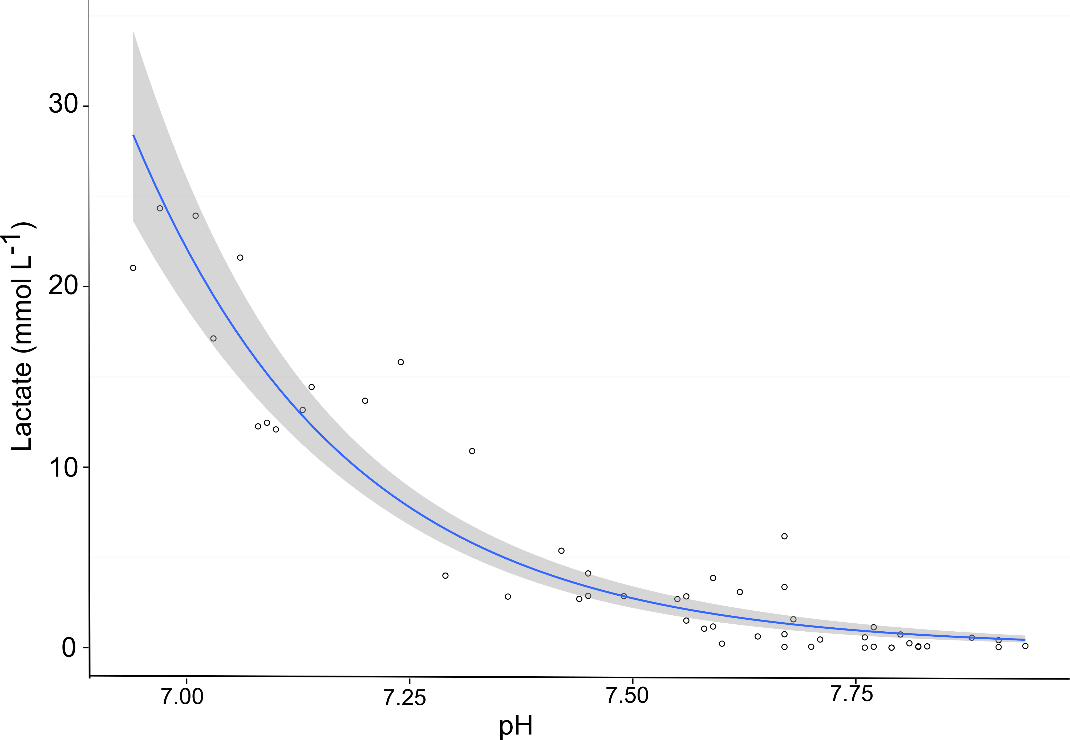


Figure S1. Relationship between haemolymph pH and lactate (mmol L-1)

Supplement: S1 Appendix — (DOCX) [file pone.0166343.s001.docx]
