# Supplementary material for: Spatial Variability in Condition of Southern Rock Lobsters (Jasus edwardsii) at the Start of the Tasmanian Fishing Season
Source: PLoS One. 2016 Nov 15;11(11):e0166343. doi: 10.1371/journal.pone.0166343 (PMC5112911; doi:10.1371/journal.pone.0166343)
Supplement: S2 Appendix — (DOCX) [file pone.0166343.s002.docx]

Supplementary material 2. Average risk of dying and associated standard errors per site

| Site | Colour | Depth | Risk of dying | SE |
| --- | --- | --- | --- | --- |
| F2 | Red | 25 | 0.84 | 0.25 |
| O2 | Red | 20 | 0.87 | 0.10 |
| O3 | Red | 20 | 0.56 | 0.27 |
| SE2 | Brindle | 88 | 0.83 | 0.11 |
| SW2 | Brindle | 99 | 1.13 | 0.16 |
| SW3 | Brindle | 84 | 0.71 | 0.20 |
